# Thyroid Involvement in Two Patients with Bannayan-Riley-Ruvalcaba Syndrome

**DOI:** 10.4274/Jcrpe.984

**Published:** 2013-12-12

**Authors:** Valentina Peiretti, Alessandro Mussa, Francesca Feyles, Gerdi Tuli, Arianna Santanera, Cristina Molinatto, Giovanni Battista Ferrero, Andrea Corrias

**Affiliations:** 1 University of Torino, Regina Margherita Children Hospital, Department of Pediatrics, Torino, Italy

**Keywords:** Bannayan-Riley-Ruvalcaba syndrome, PTEN, Thyroid, thyroid ultrasound, thyroid neoplasm

## Abstract

Bannayan-Riley-Ruvalcaba syndrome (BRRs) is an overgrowth disorder characterized by macrocephaly, pigmented maculae of the glans penis, and benign mesodermal hamartomas (primarily subcutaneous and visceral lipomas, multiple hemangiomas, and intestinal polyps). Dysmorphic features as well as delayed neuropsychomotor development can also be present. These patients have also a higher risk of developing tumors, as the gene involved in BRRs is phosphatase and tensin homologue (PTEN), and up to 30% of the patients have thyroid involvement consistent with multinodular goiter, thyroid adenoma, differentiated non-medullary thyroid cancer, or Hashimoto’s thyroiditis. Here, we report two cases of BRRs at opposite ends of its phenotypic spectrum: clinical manifestations of the first patient were more severe, while the second one showed only few signs and had no family history of the disease. Both cases developed thyroid disorders detected by thyroid ultrasound screening. We believe that it is important for clinicians, specifically pediatric endocrinologists, to know that this syndrome can appear in very subtle ways and also to be aware that thyroid nodules and intestinal polyps seem to be its most frequently encountered features.

**Conflict of interest:**None declared.

## INTRODUCTION

First described in 1971 (1), Bannayan-Riley-Ruvalcaba syndrome (BRRs, OMIM #153480) is an overgrowth disorder characterized by macrocephaly (head circumference >97th percentile), pigmented maculae of the glans penis, and benign mesodermal hamartomas, as subcutaneous and visceral lipomas, multiple hemangiomas and intestinal polyps. Neonatal macrosomia (birth weight >97th percentile), increased linear growth, dysmorphic features, joint hyperextensibility, pectus excavatum, scoliosis, and café au lait spots (2,3) are additional characteristics that can be frequently present. Face abnormalities include frontal bossing, hypertelorism, down-slanting palpebral fissures, epicanthus inversus, long philtrum with thin upper lip, and broad mouth with relative micrognathia (2,4). In addition, neuromuscular disorders are often present, encompassing myopaty of proximal muscles (60% of the patients) and delayed neuropsychomotor development of variable degree (20 to 50%) (2). In some of patients, arteriovenous malformations have also been reported (5). Intestinal polyps occur in 35-40% (4,6) of patients and they manifest in childhood with chronic anemia, diarrhea or small bowel invagination (4,7,8). The oncologic risk is typically increased with predisposition to develop intestinal, thyroidal, uterine and breast cancer. Up to 30% of BRRs patients have thyroid involvement consistent with multinodular goiter, thyroid adenoma, differentiated non-medullary thyroid cancer (9) or Hashimoto’s thyroiditis (3).

BRRs has an autosomal dominant inheritance (4), with 37% of cases caused by new mutations (10). Germline inactivating mutations of phosphatase and tensin homologue (PTEN) gene are found in approximately 60% of individuals who meet the clinical diagnosis of BRRs (11). PTEN gene is a tumor suppressor gene mapping on chromosome 10q23.3, with relevant role in the pathway of cellular proliferation, migration and apoptosis; somatic mutations have been found in several human solid tumors (4,12), explaining the increased cancer risk in BRRs. PTEN mutations are responsible for the broader definition of the PTEN hamartoma tumor syndromes (PHTS), a group of diseases also including Cowden syndrome (CS), PTEN-related Proteus syndrome, Proteus-like syndrome, and Lhermitte-Duclos syndrome. All PHTS present different PTEN mutations and some degree of clinical overlap, although they can usually be distinguished from one another (2,13).

BRRs itself is a pleiotropic syndrome and its many clinical features rarely manifest constantly in each patient; rather it manifests with a wide spectrum of phenotypic traits. There are patients with severe symptoms and patients with manifestations that can go unnoticed. Here we report two pediatric cases of BRRs well exemplifying the broad phenotypic spectrum of the disease.

## CASE REPORT A

This male patient was first seen at age 11 years, when he presented with rapidly developing obesity consistent with eating disorder and was admitted to our Institution for further investigation. The patient was born with spontaneous delivery following a 41 weeks pregnancy. There was a history of threatened abortion in the third month. At birth, the patient’s auxological parameters were all above the 97th percentile (weight: 5180 g, length: 57.8 cm, head circumference: 41.5 cm). He was reported to have suffered a clavicle fracture with sternocleidomastoid hematoma. Because of the progression of macrocephaly during the first semester of life, a brain ultrasonography (US) was performed, which showed symmetrical dilation of the cerebral ventricles. Computed tomography and magnetic resonance imaging (MRI) scan performed at that time showed normal morphology and density of cerebal ventricles, but an asymmetrical skull with volumetric reduction of the right cerebral hemisphere; proximal hyposthenia and joint hypermobility were also present. Standard karyotype was normal 46,XY.

At the age of 3 years, the patient was noted to have mild retardation in neuropsychomotor development (first words at 12 months, walking at 18 months). At this time, he developed a large lipoma in his left axilla that was surgically removed. At age 10 years, three very closely located lipomas 6 mm in diameter developed in the patient’s left hand and also one on the left side of his neck, so he again underwent surgery. Since age 5 years, a severe scoliosis developed, and in the next few years, it progressed so much, that the boy needed to be operated on three times (at 6, 12 and 16 years of age) with anterior spine fixation.

At admission, the patient was found to be at the 4th stage of pubertal development, according Tanner staging: pubertal development was reported to have been precocious, with testes enlargement occurring at 8 years of age, and progressing rapidly. Pigmented maculae on the glans penis were present. A hand and wrist X-ray that was performed to estimate the bone age (13 years 4 months) also revealed bilateral dysmorphic features in the metacarpophalangeal bones. Routine blood chemistry and hormonal studies showed normal levels for glucose and basal insulin, for thyroid stimulating hormone, free thyroxine, thyroid antibodies, adrenocorticotropic hormone and cortisol with overnight dexamethasone suppression test. The luteinizing hormone release-factor standard test also indicated a physiologic central activation. Fecal occult blood test was negative. At clinical evaluation, the thyroid gland was noted to be enlarged and US of the gland showed lobes of 40x20x15 mm diameter with nonhomogeneous echotexture, due to several hypoechoic areas of a few millimeters in diameter. Neurologic examination revealed a severe psychomotor retardation, with slow and uncoordinated movements together with proximal limbs asthenia: the MRI of the brain and spine demonstrated focal temporal pachygyria in the right lobe.

BRRs was suspected after genetic counseling, and PTEN mutation was searched in DNA extracted from peripheral blood cells. The analysis revealed a de-novo mutation c.219C>T resulting in premature stop codon.

**Figure 1:**
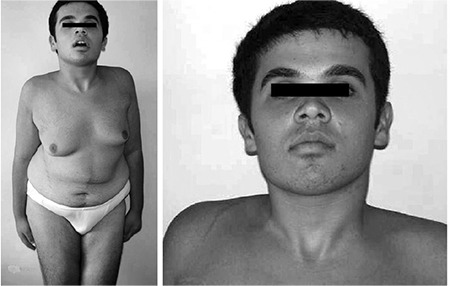
Case A at the age of 15 years. Note the severe scoliosis and typical dysmorphic features

After discharge, the patient was followed up three times a year. A physical examination, abdominal and thyroid US, endocrinological, orthopedic and dermatological consultations were performed, and fecal occult blood test and laboratory tests including blood cell count and thyroid hormone and antibody levels were assessed at each visit. At the age of 13 years, an arteriovenous malformation was detected in the left hand: it rapidly progressed and increased in size. It became necessary to perform a percutaneous embolization of the radial artery because it was very dilated (4.5 mm diameter) and both arterial and venous flux of the malformation was accelerated and turbulent. At the age of 14 years, one of the thyroid nodules increased in size, reaching 13 mm in maximum diameter: the boy therefore underwent fine-needle aspiration biopsy and cytology revealed Hurthle-cell (oxyphilic) carcinoma. Total thyroidectomy was performed and histology demonstrated a well capsulated oxyphilic adenoma without capsule invasion or metastases in the lymph nodes. Starting at age 15 years (Figure 1), the patient also underwent a neuropsychiatric evaluation at each follow-up visit, because of progressive development of the eating disorder and aggressiveness; he was treated with pimozide and delorazepam.

Now, at the age of 22, he is followed up twice yearly. Fecal occult blood test never turned positive, however, a first colonoscopy is scheduled at 23 years. Obesity is severe, with a body mass index value of 39. Every 2 years MRI of the brain is performed. The patient is on substitutive therapy with levothyroxine and assessment of thyroid hormone levels is performed regularly. Neuropsychiatric visits are continued on a monthly basis. 

## CASE REPORT B

This female patient was referred to our Department because of her suspected syndromic macrocephaly at the age of 5 years and 3 months. She was born at a gestational age of 35 weeks by caesarian section because of premature rupture of membranes. At birth, she weighed 3000 g (90th percentile) and was 50 cm long (90th percentile) with a head circumference of 33.5 cm (97th percentile). Although development was assessed as normal in her first year of life, a divergence was noted between her length (always at 97th percentile) and her head circumference, that rapidly increased to exceed the 97th percentile. Therefore, she underwent brain US several times. MRI revealed dilation of cerebral ventricles. However, her neuropsychomotor development was normal. At age 3 years, an inguinal lipoma developed and its diameter increased up to 3 cm, needing to be removed.

At physical examination at age 5 years, the patient was found to have macrocephaly (head circumference: 57 cm, >97th percentile) with frontal bossing and a couple of small café au lait spots on the abdomen. Standard karyotype was 46,XX. A possible diagnosis of BRRs presenting with mild symptomatology was suspected due to the association between macrocephaly and lipoma. The patient’s PTEN gene sequencing revealed a de-novo mutation c.635G>C, causing truncated protein.

Since the time of this diagnosis at age 5 years and 7 months, the patient is being followed up twice yearly. The follow-up visits include physical examination, orthopedic and dermatological consultations, performing a fecal occult blood test, blood cell count, dosage of thyroid hormone and antibodies, and finally thyroid and abdomen US. At age 6 years and 7 months, the dermatological assessment revealed development of multiple small subcutaneous lipomas, a few millimeters in diameter, on her chest and abdomen. Thyroid function tests were always normal with negative results for anti-thyroperoxidase and anti-thyroglobulin antibodies. At 8 years of age, abdominal US revealed mild splenomegaly with homogeneous echo texture. Two consecutive thyroid US assessments revealed a growing hypoechoic nodule that rapidly reached a size of 23x9x16 mm, involving the isthmus and left lobe. Fine-needle aspiration biopsy revealed “suspect” citology, so the patient underwent total thyroidectomy. Histology demonstrated follicular capsulated adenoma with neither invasion nor nodal involvement. Now she is on substitution therapy with levothyroxine and is followed up twice yearly with thyroid function tests. At the age of 9 years, mild right lumbar scoliosis was noticed during the orthopedic follow-up: it was thought to be due to poor posture since X-ray of the skeletal column was normal and no skeletal anomalies were detected. Search for fecal blood turned positive at 10 years of age and at the same time microcytic anemia was noticed. Esophagogastroduodenoscopy and colonoscopy showed 3 pedunculated polyps (the largest one was 15 mm in size), in the transverse and descending colons, which were all endoscopically removed. Histology revealed benign hamartomatous lesions with chronic inflammation.

**Figure 2:**
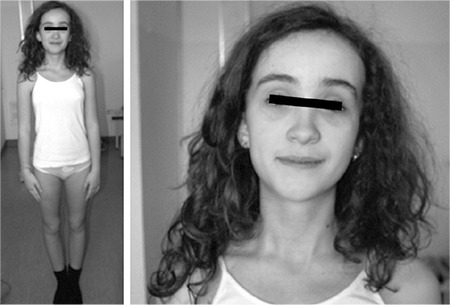
Case B at the age of 12 years. Note the macrocephaly

At present, this patient is being followed up twice yearly for her iatrogenic hypothyroidism (with thyroid hormone dosage) and intestinal polyps (with fecal occult blood test and abdomen US). Once a year, she undergoes orthopedic, dermatologic and ophthalmologic examinations and MRI of the brain is performed every two to three years. Now at the age of 12 years (Figure 2), she is developing well, has no further relapses of lipomas, nor intestinal polyps, her scoliosis does not get worse, has completely normal neuropsychomotor development with brilliant results at school and a fully satisfying quality of life.

## DISCUSSION

BRRs is a pleiotropic genetic condition, encompassing macrocephaly, lipomas, hemangiomas and intestinal polyps, skeletal malformations, and penile glans maculae in males. Neuropsychomotor development is delayed in approximately half of the patients. All these features combined in different ways may present in a particularly broad phenotypic spectrum. Currently, diagnostic criteria for BRRs are not universally shared, although the syndrome is usually diagnosed if at least 3 of the 4 major features (macrocephaly, lipomatosis, hemangiomas, and speckled penis) are found, according to Marsh and Laury et al (10,14). In some cases, presence of two features are considered sufficient (15).

We report two cases, followed up at our Institution, which well illustrate the variability of syndrome presentation, being at opposite ends of its phenotypic spectrum. Clinical manifestations of the first patient were more severe, including almost all features of BRRs, while in the second patient, macrocephaly was the sole relevant clue to diagnosis and the other features were mild and subtle. A careful work-up of this patient enabled us to detect several other features of BRRs such as follicular adenoma diagnosed by thyroid US and intestinal polyposis by fecal occult blood test and colonoscopy. Clinical features of the two patients are summarized and compared in Table 1.

Findings in patient B strengthen the need for a meticulous evaluation of the clinical manifestations of BRRs, as there may be some cases that present with very subtle features, few signs and no family history of disease. These signs need to be investigated specifically, as was the case in our girl patient. The early recognition of the condition is of paramount importance, mostly because of its oncologic implications. Actually, affected individuals with PHTS develop both benign and malignant tumors in a variety of tissues, such as breast, uterus and thyroid, and screening procedures for early cancer detection are crucial. Screening protocols are well defined for adult patients with CS, while recommendations for BRRs are less robust (16). Consistent with procedures adopted for other overlapping overgrowth/cancer predisposition syndromes (17), pediatric patients with BRRs should be followed and subjected to a screening including complete blood count, urinalysis, abdominal and thyroidal US twice yearly. It is also important to detect intestinal polyps through fecal occult blood test, eventually followed by colonoscopy. Due to the higher risk of developing intracranial tumors, periodic brain MRI is also indicated.

Of particular interest to pediatric endocrinologists is the involvement of the thyroid gland in patients with BRRs. In fact, both of our cases developed thyroid neoplasm. Follicular and papillary carcinomas are frequently encountered in BRRs and are reported to occur in almost 70% of cases (14). Besides malignancies, multiple adenomatous thyroid disease and lymphocytic thyroiditis are also frequently encountered in BRRs patients. In previous series of thyroid nodules in BRRs, mean age at diagnosis was 14 years and our report is consistent with this finding, supporting the need for extend screening recommendations to the pediatric ages, as also suggested by other authors (16). Follicular thyroid neoplasms are important features of BRRs, as well as of other PHTS: these kinds of differentiated thyroid cancers are frequently multicentric and are believed to arise from follicular adenomas (14,16).

To our knowledge, Hurthle-cell adenoma or carcinoma has never been reported in PHTS and our case represents the first report. Although both our patients developed thyroid neoplasm and underwent total thyroidectomy, in both cases histology revealed the presence of benign neoplasm in spite of suspect cytology. Actually, the oxyphilic and follicular cytology do not allow to completely distinguish between benign and malignant nodules and prophylactic surgery is recommended (18,19,20,21). However, contrarily to recommendations for other syndromes with thyroid cancer predisposition such as multiple endocrine neoplasia syndromes, thyroidectomy is not employed in BRRs patients without nodules (18).

In conclusion, these case reports demonstrate the very broad and variable phenotypic spectrum in presentation of BRRs. It is important to bear in mind that, besides complete and severe cases, BRRs can be subtle in presentation. Clinicians, and specifically pediatric endocrinologists, are advised to have a high diagnostic suspicion for BRRs to prompt the diagnosis of thyroidal nodules and of intestinal polyps, which are among the frequently encountered features of the syndrome. 

## Figures and Tables

**Table 1:**
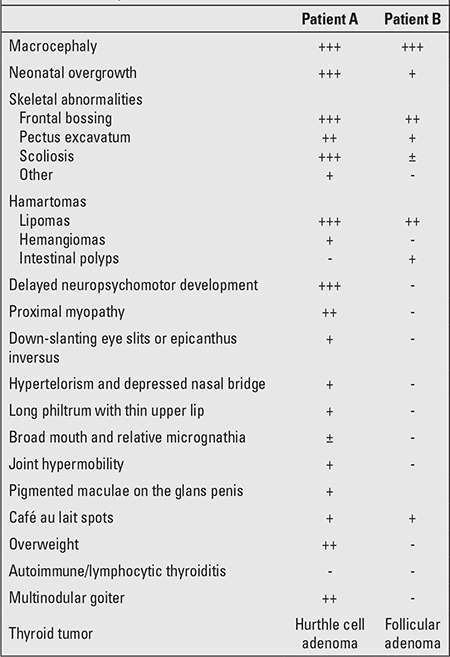
Clinical features of Bannayan-Riley-Ruvalcaba syndrome (BRRs) in the two patients
